# Barriers to Biomarker-Guided Advanced Breast Cancer Care in India: Diagnostic, Economic, and Implementation Challenges

**DOI:** 10.7759/cureus.116761

**Published:** 2026-09-22

**Authors:** Sandeep Jasuja, Rona Joseph P, Rajeev Gupta, Prashant Sada

**Affiliations:** 1 Oncology, Sawai Man Singh (SMS) Medical College, Jaipur, IND; 2 Medical Oncology, Regional Cancer Centre, Trivandrum, IND; 3 Oncology, State Cancer Institute, Jammu, IND; 4 Medical Affairs, AstraZeneca Pharma India Ltd, Bangalore, IND

**Keywords:** breast cancer, diagnosis, human epidermal growth factor receptor-2, insurance policy, treatment paradigm

## Abstract

Breast cancer is the leading cancer among women in India, with a high proportion of cases presenting at advanced or metastatic stages. While precision oncology has transformed global breast cancer care through biomarker-guided therapies, including those targeting human epidermal growth factor receptor 2 (HER2)-positive, HER2-low, and hormone receptor (HR)-positive/HER2-negative subtypes, India continues to face substantial gaps in diagnosis, access, and clinical implementation of these advances. These disparities indicate persistent gaps in access to tailored treatments and the effective implementation of precision oncology in India.

This review outlines the clinical, economic, and psychosocial consequences of limited access to precision oncology for breast cancer in India. Limited availability and inconsistent biomarker testing (e.g., HER2, HR, BRCA1/2), high out-of-pocket costs, fragmented referral systems, and poor adherence to international treatment guidelines contribute to suboptimal care and avoidable morbidity. Limited access to biomarker-guided therapies has been associated with higher toxicity, diminished quality of life, and greater psychosocial distress.

National health initiatives aimed at expanding insurance coverage and enhancing digital health infrastructure have the potential to improve access through policy reforms; however, substantial financial and infrastructural barriers persist. Evidence suggests that standardization of diagnostic protocols, broader insurance coverage for therapies such as cyclin-dependent kinase 4/6 (CDK4/6) inhibitors and trastuzumab deruxtecan (T-DXd), and expanded continuing medical education could address existing gaps in care. Emerging evidence suggests that patient advocacy initiatives, AI-assisted pathology tools, and regionally integrated care models may help improve diagnostic consistency and address urban-rural disparities in access to care. Alignment with global precision oncology standards requires coordinated action across public health policy, clinical practice, and diagnostic infrastructure. Addressing these barriers may improve the delivery of biomarker-guided care and contribute to better outcomes for patients with breast cancer in India.

## Introduction and background

Breast cancer is among the most frequently diagnosed malignancies in females globally. As per estimates, breast cancer accounted for 26.6% of all female cancers in 2022, with 192,020 new cases reported nationwide [1]. Despite advances in awareness, imaging, and treatment, the burden of late-stage presentation remains disproportionately high in India. Multiple studies indicate that 57-60% of breast cancer cases in India are diagnosed at stage III or IV [2-4], underscoring the high burden of advanced-stage presentation. In contrast, fewer than 10% of patients in most high-income countries present with stage IV disease at diagnosis [5], reflecting the benefits of established early detection and screening programs.

The delay in diagnosis of breast cancer can impact survival outcomes [6]. In India, the median overall survival (OS) for patients with advanced or metastatic breast cancer (mBC) ranges from 18 to 32 months, depending on the disease setting and access to treatment [7]. This is significantly lower than that reported in high-income countries, where median OS for mBC is generally higher, particularly among patients with human epidermal growth factor receptor 2 (HER2)-positive disease, for whom median OS can approach or even exceed five years in some subgroups. This survival advantage may reflect broader access to modern targeted therapies, timely diagnosis, and well-established healthcare infrastructure [8-10]. Reflecting this disparity, the five-year survival rate for breast cancer in high-income countries exceeds 90%, compared with 66% in India [11-13]. Furthermore, stage-specific analyses also demonstrate lower five-year survival in India for localized disease (81.0% versus 100%), regional disease (65.5% versus 87.5%), and distant/metastatic disease (18.3% versus 33.8%) relative to reported US population-based estimates [14,15].

The recognition of breast cancer as a biologically heterogeneous disease has led to the molecular classification of distinct intrinsic subtypes: luminal A, luminal B, HER2-enriched, and triple-negative breast cancer (TNBC), each with distinct prognostic profiles and therapeutic implications. Among these, HER2-positive breast cancer, defined by HER2 gene amplification or protein overexpression, accounts for 12% to 19% of Indian cases, although estimates vary based on testing methodologies and geographic heterogeneity [16]. In the absence of HER2-targeted therapy, HER2-positive breast cancers are associated with a significantly poorer prognosis compared with luminal (HR-positive/HER2-negative) subtypes [17].

The introduction of HER2-targeted therapies, such as trastuzumab, pertuzumab, and later-generation tyrosine kinase inhibitors, has substantially improved outcomes for patients with HER2-positive disease. However, recent refinements in HER2 classification have introduced the concepts of HER2-low (immunohistochemistry (IHC) 1+ or IHC 2+/in situ hybridization-negative) and HER2-ultralow (IHC 0 with faint/incomplete membrane staining). Thus, the evolving relevance of HER2-low tumors emphasizes the importance of standardized HER2 testing and reporting to guide appropriate therapy selection [18,19].

Emerging real-world data from India suggest a relatively high prevalence of HER2-low breast cancer (around 60%) [20]. This finding underscores the pressing need for standardized, high-fidelity HER2 testing to accurately distinguish low and ultralow expression levels and ensure appropriate patient selection for HER2-targeted therapies.

Despite these therapeutic advances, implementation challenges remain significant in India. Inadequate public health literacy regarding breast cancer symptoms, risk factors, and the availability of screening services, along with cultural stigma and emotional barriers such as fear, denial, and misconceptions, contributes to delayed diagnosis and intervention [21,22]. In addition, inequitable access to diagnostic infrastructure and systemic treatments further limits the real-world impact of precision oncology. Biomarker testing is frequently suboptimal due to high out-of-pocket costs, lack of uniform pathology protocols, and limited availability of trained personnel [23-25]. As a result, many patients eligible for HER2-directed therapies remain unidentified or undertreated [23,26].

Hormone receptor-positive/HER2-negative (HR+/HER2−) breast cancer represents the most common molecular subtype in India, comprising approximately 33% to 48% of cases [16]. Although endocrine therapy remains the mainstay of treatment, access to cyclin-dependent kinase 4/6 (CDK4/6) inhibitors remains limited in India due to financial and reimbursement constraints [27,28]. This often results in earlier initiation of chemotherapy, which can lead to higher toxicity, suboptimal outcomes, and poor quality of life [29-32]. Furthermore, molecular profiling and biomarker reassessment during disease progression are not routinely performed, limiting opportunities for personalized sequencing and therapeutic optimization [33-35]. Addressing these limitations is essential, as HR+/HER2− disease constitutes a significant segment of the breast cancer population with the potential to benefit from targeted approaches.

This review aims to evaluate the current landscape of biomarker-guided treatment of advanced and metastatic breast cancer in India, including HER2-positive, HER2-low, HER2-ultralow, and HR-positive subtypes. It further identifies key systemic gaps in diagnostics, testing standardization, and access to novel therapies. Addressing these gaps through nationwide implementation of quality-controlled biomarker testing, equitable access to HER2-targeted agents, and integrated oncology care models is critical to advancing precision oncology and improving survival outcomes for Indian patients with breast cancer.

## Review

Methodology

This article presents a narrative review of published literature examining the diagnosis, treatment patterns, and real-world implementation of precision oncology in breast cancer in India, with particular emphasis on advanced and metastatic disease. The review focuses on clinically relevant subtypes, including HER2-positive disease, HER2-low and HER2-ultralow categories, and HR-positive/HER2-negative breast cancer. Literature searches were performed using PubMed and Google Scholar up to August 31, 2025, with additional sources identified through manual review of reference lists from key publications. Search terms included combinations of “breast cancer,” “India,” “precision oncology,” “biomarker testing,” “HER2,” “HER2-low,” “HER2-ultralow,” “HR-positive breast cancer,” “CDK4/6 inhibitors,” “BRCA,” “targeted therapy,” “metastatic breast cancer,” “psychosocial outcomes,” and “oncology workforce.”

Eligible sources included peer-reviewed original research articles, observational studies, registry-based analyses, systematic and narrative reviews, clinical practice guidelines, governmental reports, policy documents, and conference abstracts relevant to biomarker-guided breast cancer care. The review primarily considered literature published over the past 10-15 years, with greater emphasis on evidence generated since 2018 to reflect contemporary diagnostic frameworks, treatment strategies, and health-system challenges. Sources were selected based on their relevance to biomarker testing, targeted treatment access, implementation barriers, clinical outcomes, and healthcare delivery in India. Publications in English were reviewed.

Particular emphasis was placed on studies reporting Indian data and real-world experience, while pivotal phase III clinical trials and relevant clinical guidelines, including those from the Indian Council of Medical Research (ICMR), American Society of Clinical Oncology/College of American Pathologists (ASCO/CAP), National Comprehensive Cancer Network (NCCN), and European Society for Medical Oncology (ESMO), were reviewed to contextualize treatment efficacy and biomarker-guided decision-making. Additional sources such as cancer registry reports, governmental publications, regulatory approvals, and public insurance scheme documentation referenced in the article were also examined. Evidence was synthesized narratively to provide an overview of the clinical, diagnostic, economic, and health-system factors affecting biomarker-guided breast cancer care in India. No quantitative pooling, meta-analysis, or formal risk-of-bias assessment was undertaken, consistent with the narrative review design.

Current diagnostic and treatment landscape of advanced/metastatic breast cancer in India

Real-world evidence from India indicates substantial implementation barriers in terms of access to diagnostics, timely treatment initiation, affordability of targeted therapies, and healthcare infrastructure limitations in breast cancer care. A tertiary-center study from India reported that approximately 57% of eligible patients with HER2-overexpressing or amplified breast cancer received HER2-targeted therapy, reflecting improved access to trastuzumab-based treatment; however, further efforts are required to achieve universal access [36].

Diagnostic variability contributes to these gaps: multicenter analyses showed 2.5%-5.7% [37] and 8.4% [38] discordance in HER2 testing between local and central laboratories, attributed to inconsistencies in IHC scoring, interpretation, and sample handling [18,23,26]. While advanced imaging and molecular diagnostics such as IHC, fluorescence in situ hybridization (FISH), and next-generation sequencing (NGS) are increasingly available in major Indian cities, access remains limited in rural and semi-urban areas. This geographic disparity contributes to delayed diagnosis and suboptimal staging and restricts appropriate patient stratification for targeted therapies [35,39,40]. While estrogen receptor (ER), progesterone receptor (PR), HER2, and breast cancer gene mutations (BRCA1 and BRCA2) testing are common in tertiary centers, coverage of emerging biomarkers such as PD-L1 [41] and NGS-dependent mutations remains low [42,43].

The management of mBC has transitioned from empirical chemotherapy toward biomarker-driven precision oncology [44-47]. This shift, supported by advances in molecular subtyping, targeted agents, and treatment sequencing, has improved survival globally, with median OS now exceeding five years in high-income settings [45,46,48]. In contrast, systemic and infrastructural constraints in India continue to limit equitable integration of these advancements [49,50].

Several pivotal trials have redefined the therapeutic landscape of mBC. The CLEOPATRA trial demonstrated that dual HER2 blockade with trastuzumab and pertuzumab significantly improved OS in HER2-positive mBC [10]. Similarly, CDK4/6 inhibitors such as palbociclib, ribociclib, and abemaciclib have substantially prolonged progression-free survival (PFS) when combined with endocrine therapy in HR+/HER2− mBC, as evidenced by PALOMA, MONALEESA, and MONARCH trial data [29,31,32,51]. As shown in DESTINY-Breast04, trastuzumab deruxtecan (T-DXd) improves survival in HER2-low mBC, which is particularly relevant for Indian patients given the high prevalence of HER2-low disease [20,52]. Additionally, in the second-line setting, T-DXd has outperformed trastuzumab emtansine (T-DM1) in HER2-positive disease, as shown in the DESTINY-Breast03 trial [53].

As discussed earlier, the emergence of HER2-low as a targetable subgroup expands the potential for novel agents in India's treatment landscape [19]. T-DXd has shown benefit in this population, with DESTINY-Breast04 reporting a median PFS of 9.9 months compared with 5.1 months for the physician's choice of chemotherapy (p<0.001) [52]. This finding is particularly relevant in India, where HER2-low tumors contribute considerably to all breast cancers [54,55]. While T-DXd's Central Drugs Standard Control Organization (CDSCO) approval for these subtypes is a notable milestone [56], its adoption is still limited by cost and infrastructure constraints.

Emerging Indian breast cancer data suggest a younger median age at presentation (40-54 years) [57-59] than that reported in several studies from high-income countries (61-63 years) [60,61]. These epidemiological and molecular differences suggest that breast cancer in India may have distinct biological characteristics. This highlights the need for India-specific clinical validation of global precision oncology approaches to ensure their relevance, effectiveness, and equitable application in the local population.

India is positioned to benefit significantly from advances in precision oncology, especially in subgroups such as HER2-low and HR+/HER2− breast cancer. However, persistent disparities in diagnostic capabilities, treatment access, and financial protection remain major barriers. A coordinated, evidence-informed national strategy [62], incorporating standardized biomarker testing and quality-assurance measures [23], regional capacity building [63], and expanded reimbursement mechanisms [64], is essential to ensure that recent therapeutic innovations translate into improved outcomes for all patients, regardless of geography or socioeconomic status. Table 1 provides a consolidated overview of key Indian studies supporting these gaps across biomarker testing, targeted therapy access, and precision oncology implementation.

**Table 1:** Key Indian evidence on biomarker testing, targeted therapy access, and precision oncology implementation in advanced breast cancer BRCA1/2: breast cancer genes 1 and 2; CDK4/6: cyclin-dependent kinases 4 and 6; CGP: comprehensive genomic profiling; CNS: central nervous system; EQA: external quality assessment; ER: estrogen receptor; FISH: fluorescence in situ hybridization; HER2: human epidermal growth factor receptor 2; HR: hormone receptor; IHC: immunohistochemistry; Ki-67: Ki-67 proliferation index; mBC: metastatic breast cancer; OS: overall survival; PFS: progression-free survival; PI3K: phosphoinositide 3-kinase; PIK3CA: phosphatidylinositol-4,5-bisphosphate 3-kinase catalytic subunit alpha; PR: progesterone receptor. Data from References [7,20,23,33,34,36,65,66,67].

Study	Population	Biomarker/Focus Area	Key Findings
Nair et al. (2022)	N = 4,717 (2016 cohort)	HER2 testing and access to HER2-targeted therapy	Estimated HER2-positive tumors: 928. Trastuzumab received by 474/831 eligible patients (57.03%). HER2-targeted therapy use increased from 8.61% (2008) to 57.03% (2016). 303/641 HER2 IHC 2+ cases did not undergo FISH testing.
Gogia et al. (2019)	N = 375 (patients with upfront mBC)	HR/HER2 status and HER2-targeted therapy utilization	HER2-positive patients: 145. Received targeted therapy: 78 (53.8%). Median PFS among HER2-positive patients: 19.7 versus 9.3 months (targeted therapy versus no targeted therapy). Median OS among HER2-positive patients: not reached versus 24.1 months.
Gogia et al. (2019)	N = 103 (patients with recurrent mBC and paired primary/metastatic biomarker assessments)	ER, PR, and HER2 biomarker discordance at metastasis	ER discordance: 22/103 (21.3%); PR discordance: 30/103 (29.1%); HER2 discordance: 16/103 (15.5%). Treatment modified in 12/21 patients with receptor gain.
Shet (2017)	Review of ER, PR, and HER2 testing practices in India	Biomarker testing quality and standardization	Indian ER/PR positivity rates ranged from 32.56% to 70.0% across studies. HER2 positivity ranged from 16.7% to 46.37% across studies. Erroneous ER/PR results were largely attributed to fixation and antigen retrieval issues. HER2 inaccuracies were linked to non-validated antibodies and interpretative errors.
Goel et al. (2024)	N = 80 (patients with HR-positive mBC screened for PIK3CA mutations)	PIK3CA mutations and access to PI3K-targeted therapy	PIK3CA mutations detected in 26/80 patients (32.5%). Most common mutation: H1047R (8/26). CNS involvement at relapse in 6/26 (23.1%) PIK3CA-mutated cases. Only 8/26 patients with PIK3CA-mutated disease received alpelisib plus fulvestrant.
Shanthala et al. (2024)	N = 51 (patients with paired primary and recurrent/mBC samples)	Biomarker discordance (ER, PR, HER2, Ki-67) between primary and recurrent/metastatic disease	ER conversion: 5/51 (9.8%). PR conversion: 11/51 (21.56%). HER2 conversion: 3/51 (5.88%). Ki-67 gain observed in 25/51 (49.0%) patients.
Gogia et al. (2023)	N = 1,932 (breast cancer patients with available clinicopathologic data)	HER2-low breast cancer prevalence	HER2-high: 600/1,932 (31.0%). HER2-low prevalence: 798/1,332 (59.9%) among HER2-low/HER2-0 tumors. HR-positive among HER2-low: 640/798 (80.0%). HR-negative among HER2-low: 158/798 (19.8%). HER2-low prevalence among HR-negative cases: 158/532 (29.69%).
Agrawal et al. (2021)	N = 188 (HR+/HER2− mBC patients receiving palbociclib)	Access and outcomes of biomarker-guided targeted therapy (CDK4/6 inhibitor)	First-line palbociclib: 115/188 (61.2%) patients. Second-line palbociclib: 73/188 (38.8%) patients. Median PFS: 20.2 months versus 12.0 months in first-line versus second-line use (p < 0.0001). Objective response rate: 80.0% versus 47.9% in first-line versus second-line use.
Mathew et al. (2022)	N = 221 (patients with advanced non-hematologic malignancies, including 34 patients with breast cancer, undergoing comprehensive genomic profiling in India)	Comprehensive genomic profiling and precision oncology implementation	Actionable alterations identified in 96/221 patients (43%). Targeted therapy received by 21/221 patients (10%). Clinical benefit observed in 9/221 patients (4%). Exceptional response observed in 2/221 patients (1%).

Consequences of non-tailored treatment in advanced or metastatic breast cancer

Despite global advancements in precision oncology, access to biomarker-guided, individualized treatment for advanced or metastatic breast cancer (mBC) remains suboptimal in India [35,36,65]. This gap in personalized care contributes to a cascade of adverse clinical, economic, and psychosocial outcomes for patients navigating treatment in resource-limited settings.

Clinical Consequences

Advances in targeted therapies have substantially improved outcomes in specific molecular subgroups of mBC, including HER2-positive, HER2-low, HR-positive/HER2-negative, and BRCA-mutated disease. In the absence of these targeted therapies, patients with HER2-positive mBC experience significantly poorer clinical outcomes, including reduced response rates and shorter OS [10]. Patients with HR-positive/HER2-negative mBC derive substantial clinical benefit from CDK4/6 inhibitor-based therapy, and lack of access to these agents may result in earlier disease progression and inferior outcomes [31,32,51]. Similarly, patients with germline BRCA-mutated mBC experience poorer outcomes when effective targeted therapies such as poly(ADP-ribose) polymerase (PARP) inhibitors are unavailable [68]. Comparable benefits have been reported with other targeted treatment approaches in mBC. Dual HER2 blockade with trastuzumab and pertuzumab improves OS in patients with HER2-positive mBC [10]. Similarly, CDK4/6 inhibitors have nearly doubled PFS in HR+/HER2− patients [29,51]. Notably, T-DXd significantly improves survival in patients with HER2-low mBC [52]. This is particularly relevant in India, where recent evidence shows a high prevalence of HER2-low disease [20], and emerging data suggest the existence of a distinct HER2-ultralow subgroup, warranting further exploration [18,19]. Despite such progress, access to these therapies remains inconsistent in low- and middle-income countries, including India. A study reported that 57% of HER2-positive patients in India received HER2-targeted therapy [36], highlighting persistent access gaps and missed opportunities for improved survival. Limited access to advanced biomarker testing methods such as FISH and NGS in India, along with a lack of standardization, often leads to delayed or inaccurate results and misclassification of tumor subtypes [23,35,42,43]. This can cause eligible patients to be overlooked for appropriate targeted therapies such as T-DXd [18,19]. In HR+/HER2− mBC, the absence of routine endocrine resistance assessment and limited availability of CDK4/6 inhibitors frequently result in premature or unnecessary use of chemotherapy, ultimately compromising patient outcomes [69]. Furthermore, the lack of integrated multidisciplinary teams (MDTs) leads to fragmented care and missed nuances in biomarker interpretation and treatment sequencing, often resulting in suboptimal regimens [70].

The clinical consequences are significant. Indian real-world data indicate a median OS of just 18 to 31.7 months among patients with upfront mBC, depending on subtype and treatment access [7]. Contemporary cohorts from high-income settings have reported substantially longer survival outcomes following the integration of targeted therapies and improved treatment sequencing [44-47,71]. For example, a population-based study from the Netherlands reported a median OS of 57.3 months among patients with de novo HER2-positive mBC diagnosed during 2016-2017 [71]. Five-year survival among patients with mBC in India remains approximately 22% [7], whereas global benchmarks typically exceed 30% [72] and, in some countries, surpass 40% [44,45,48,73]. Patients who receive trastuzumab-containing regimens report improved health-related quality of life, including less fatigue, better physical and role functioning, and reduced emotional and social impairment, relative to those receiving chemotherapy alone. This suggests that inadequate access to such targeted therapies may translate into worse patient-reported outcomes (PROs), although direct studies comparing access-based differences are lacking [74]. These challenges are especially prevalent in rural areas, where fragmented referral systems and limited specialized oncology infrastructure lead to treatment delays and, despite a 20% lower incidence, result in higher mortality rates compared with urban populations [50].

Inappropriate treatment selection, such as administering chemotherapy when targeted therapy could be more effective, increases preventable toxicity and adversely affects patient outcomes. These issues are often compounded by systemic challenges in cancer care delivery in India, including inconsistent implementation of clinical guidelines and disparities in follow-up practices, which may contribute to suboptimal outcomes and compromise patients' quality of life (QoL) [25,75].

Bridging all these gaps requires policy-level interventions, improved diagnostic infrastructure, and equitable access to evidence-based therapies.

Economic Consequences

Access to targeted therapies in India remains constrained by high out-of-pocket expenditure and reimbursement gaps, despite incremental improvements in public sector availability [76,77]. A prospective study reported a mean out-of-pocket cost of ₹186,461 (~US $2,550), constituting 72.2% of total treatment expenses, with only 8% of patients receiving full reimbursement [78].

These financial challenges contribute significantly to clinical and economic consequences. Delayed or inadequate access to therapies, such as CDK4/6 inhibitors, HER2-targeted agents, and antibody-drug conjugates (ADCs), is associated with disease progression, premature mortality, and treatment-related disability. National estimates indicate that productivity losses due to breast cancer exceed ₹12,000 crore (US $1.5 billion) annually, primarily affecting younger, working-age women [24,79].

Although public insurance schemes like Ayushman Bharat Pradhan Mantri Jan Arogya Yojana (AB-PMJAY) provide partial coverage, reimbursement for newer therapies remains inconsistent. The cost of targeted therapies continues to pose a significant barrier to access in India, particularly in the absence of comprehensive reimbursement mechanisms and financial support systems [64,76,77]. Studies among patients with breast cancer in India have reported substantial financial hardship, with many households relying on distress financing, including loans, borrowings, and the sale of assets, to meet treatment-related expenses [78,80].

Limited access to affordable and timely biomarker testing further compounds the economic burden. Inconsistent coverage of initial and confirmatory diagnostics, particularly for HER2 and HR assessment, results in delayed treatment and repeat testing, often requiring out-of-pocket payment [26,78,81]. These challenges are more pronounced in rural and tier-2 regions, where public infrastructure remains under-resourced and private alternatives are cost-prohibitive [25,39,50,78,82].

From a health systems perspective, timely integration of precision oncology can reduce long-term costs by improving PFS, minimizing ineffective treatment cycles, and reducing hospitalizations [28,83]. For example, CDK4/6 inhibitors have demonstrated efficacy in HR+/HER2- advanced breast cancer, while trastuzumab deruxtecan has improved progression-free survival and OS in previously treated HER2-low mBC [29,51,52].

Investing in accessible, biomarker-guided therapies and diagnostics is therefore not only clinically essential but also economically justified. Policy reforms aimed at price regulation, broader insurance coverage, and diagnostic standardization are critical to alleviating the economic burden and ensuring equitable access to precision care.

Psychosocial Consequences

Psychological distress is highly prevalent among patients with breast cancer in India, with depression, anxiety, and fear of disease progression often intensified by visible side effects and loss of autonomy. Studies indicate that up to 60% experience depression, 73% experience anxiety, and over 80% report stress symptoms, with rates notably higher among rural and low-income populations [84-86].

While a cancer diagnosis itself is distressing, psychosocial burdens may be greater among patients who do not receive personalized treatment approaches. Chemotherapy, often the default in the absence of targeted therapies [87], is associated with adverse effects such as alopecia, fatigue, gastrointestinal toxicity, neuropathy, and premature menopause, all of which impair body image, emotional well-being, and treatment adherence. Chemotherapy in women with breast cancer is strongly associated with increased fatigue, depression, and anxiety, together with reduced QoL during and after treatment [88]. The unpredictability of outcomes with non-targeted therapies and frequent treatment failures may further compromise emotional stability and increase caregiver burden [85,89].

By contrast, biomarker-directed therapies have demonstrated clinical benefit in appropriately selected patient populations and may offer more predictable treatment pathways and favorable tolerability profiles, fostering better psychological resilience. To improve outcomes, a coordinated approach integrating biomarker-guided therapy, psychosocial care, and culturally sensitive patient education is essential. Psychological distress is widespread among patients and has been reported alongside challenges related to lack of tailored treatment [89].

In the absence of tailored treatments, the unpredictability of outcomes with non-targeted therapies and higher toxicities may adversely affect well-being, exacerbate psychosocial distress, and contribute to reduced treatment adherence and poorer outcomes [89].

Gaps in implementation of targeted therapy and standard guidelines in India

Despite advancements in molecular profiling and the availability of targeted therapies, the integration of precision oncology in breast cancer care across India remains variable. These inconsistencies stem primarily from systemic challenges, including limited diagnostic capacity, inadequate healthcare infrastructure, workforce disparities, and gaps in policy implementation. The lack of standardized, India-specific clinical guidelines further worsens these challenges, leading to inconsistent clinical decision-making and variability in care delivery. Addressing these barriers is critical to translating scientific progress into equitable improvements in patient outcomes.

Diagnostic and Testing Limitations

Reliable identification of actionable biomarkers is foundational to precision medicine. However, inconsistencies in HER2 testing continue to undermine treatment accuracy in India. Studies have highlighted ongoing challenges in achieving standardized HER2 testing across Indian laboratories, including variability in testing practices, interpretation, and quality-assurance processes [23]. These inconsistencies affect eligibility for therapies such as T-DXd, which require precise stratification of HER2-low and HER2-ultralow subtypes.

Access to comprehensive biomarker testing, including IHC, FISH, and NGS, is largely concentrated in urban tertiary centers [26]. In an Indian multicenter study, only 10% of patients with advanced cancer received targeted therapy based on comprehensive genomic profiling, and just 4% derived clinical benefit [66]. Genetic tests such as BRCA1/2 mutation panels remain cost-prohibitive and logistically inaccessible in non-urban regions, limiting their routine use in clinical decision-making. The lack of external quality-assurance programs and standardized protocols further contributes to diagnostic variability [23].

Geographic and Workforce Disparities

There is a pronounced urban-rural divide in oncology care. India’s population is predominantly rural (70%), yet approximately 95% of cancer care facilities are located in urban areas. There is a pronounced shortage of oncologists in rural regions, as the majority of graduate doctors and, by extension, cancer specialists are concentrated in cities [4,90]. As a result, patients in underserved areas face delays in diagnosis, fragmented referral pathways, and limited access to targeted therapies. The oncologist-to-patient ratio in some parts of India remains approximately 1:2000 [91]. Oncologists in India reported a median of 475 new consultations annually compared with 175 in high-income countries [92]. This disparity is further compounded by insufficient availability of trained pathologists [93], limited exposure to newer biomarkers [39], and gaps in continuing medical education [94,95].

Policy Barriers and Financial Constraints

Although CDSCO has approved several novel agents, delays in market entry remain a concern. There is often a substantial lag between international regulatory approvals and the availability of novel oncology therapies in India, which can hinder timely access to emerging treatment options [96]. Although AB-PMJAY does provide reimbursement for targeted therapies [64], the extent of coverage appears variable [76]. In a hospital-based analysis of 4,844 settled AB-PMJAY claims, medical oncology accounted for 37.45% of all claims [97]. Broader reimbursement gaps, such as a 43% shortfall for breast cancer, highlight persistent limitations in equitable access [76].

As outlined previously, high out-of-pocket costs for targeted therapies like T-DXd and CDK4/6 inhibitors remain a major barrier to access. These costs lead to treatment abandonment and suboptimal care, particularly for patients from lower-income and rural backgrounds. Financial burden affects nearly 60% of patients with breast cancer, with many borrowing money or liquidating assets to continue treatment [80].

Guideline Adoption and Clinician Awareness

Limited awareness and adoption of international treatment guidelines, such as those from the NCCN or ESMO, further contribute to practice variability. A real-world study from India involving 415 patients with breast cancer showed that treating physicians complied with NCCN guidelines for NGS testing in only 47.95% of eligible cases. Notably, undertreatment accounted for 50.36% of cases, highlighting missed opportunities for precision oncology despite affordability [43]. The rapidly evolving landscape of HER2-low classification and HER2-directed therapies [18,19] underscores the need for continued medical education and dissemination of updated clinical guidance. While institutions like Tata Memorial Center and All India Institute of Medical Sciences (AIIMS) have established continuing medical education (CME) initiatives, outreach to clinicians in tier-2 and rural centers remains inadequate [47,63].

Strategies to advance precision oncology in India

The promise of precision oncology in breast cancer cannot be realized in India without systemic reform. Diagnostic variability, workforce inequity, financial challenges, and fragmented policy implementation continue to obstruct access to life-extending therapies. Bridging these gaps will require harmonized efforts across public health systems, regulatory agencies, clinicians, and civil society. Ensuring that molecularly guided treatment is accessible to all, regardless of geography or income, must be a national imperative to reduce disparities and improve survival in mBC. While global advances in the treatment of mBC have been significant, the following integrated interventions are proposed to align India’s cancer care ecosystem with international standards while adapting to local constraints.

Policy and Financing Reforms

In India, a combination of national, state-level, and private insurance schemes provides financial protection for breast cancer treatment, including metastatic disease. At the forefront is AB-PMJAY, which offers annual coverage of ₹5 lakh per family for secondary and tertiary care, including oncology services [64,98]. The scheme encompasses packages for chemotherapy, surgery, and targeted therapies; however, access to high-cost drugs often requires pre-authorization and may be constrained by reimbursement caps. While AB-PMJAY currently does not comprehensively cover advanced therapies such as CDK4/6 inhibitors, trastuzumab, pertuzumab, and T-DXd, expanding its formulary to include these treatments is essential to improving equitable access to advanced care [36,52,64].

Complementing AB-PMJAY, state-sponsored schemes such as the Chief Minister’s Comprehensive Health Insurance Scheme (CMCHIS) in Tamil Nadu and Aarogyasri in Andhra Pradesh offer similar coverage limits and include provisions for advanced cancer therapies [99,100]. A multi-state study revealed that patients enrolled in government insurance programs were 36% more likely to initiate treatment within 30 days of diagnosis, with the most significant improvements observed in breast and head and neck cancers [101]. These findings highlight the critical role of public insurance in reducing treatment delays and enhancing access to timely care.

In the private sector, cancer-specific insurance plans may provide stage-based financial support for treatment, including coverage for selected advanced therapies. These plans explicitly cover targeted therapies, immunotherapy, and hormonal therapy under their advanced treatment benefits. Additionally, the Insurance Regulatory and Development Authority of India (IRDAI) mandates that all insurers include modern treatments, such as oral chemotherapy and targeted therapy, within the scope of standard health insurance policies [102].

Together, these insurance mechanisms represent a vital framework for reducing the economic burden of cancer care and ensuring that therapeutic advances translate into improved outcomes for patients across India’s diverse healthcare landscape.

Price regulation mechanisms, such as those implemented by the National Pharmaceutical Pricing Authority (NPPA), and the introduction of cost ceilings for essential oncology drugs could reduce the economic burden and improve adherence. Oncologists can play a pivotal role in shaping policy by advocating for the inclusion of novel therapies in national formularies, contributing to cost-effectiveness evaluations, and engaging with regulatory bodies [76]. For example, Indian clinician-researchers have historically contributed to price negotiations and public sector access for trastuzumab and other essential cancer drugs [47]. Additionally, fostering public-private partnerships (PPPs) can enhance service delivery through subsidized diagnostics, regional chemotherapy centers, and cancer units in underserved regions. Earlier PPPs under the Rashtriya Swasthya Bima Yojana (RSBY) demonstrated improvements in service availability, and similar models are being extended in cancer care today [24,82]. In India, pharmaceutical access programs are increasingly recognized and supported by official policies. Patient Assistance Programs (PAPs), including price negotiation and patient support schemes, have helped reduce costs and improve the availability of innovative cancer therapies. These initiatives play a vital role in expanding equitable access to advanced treatments, particularly in resource-limited settings. Strengthening the role of patient advocacy groups in treatment navigation, psychosocial counseling, and connecting patients to financial support programs is an underutilized yet impactful strategy. These groups can act as intermediaries between patients and institutions, especially in resource-constrained regions [103].

Diagnostic Infrastructure and Standardization

Timely and accurate diagnosis is the cornerstone of precision oncology. To reduce interlaboratory variability and ensure consistency, HER2, HR, and BRCA testing protocols should be standardized in accordance with ASCO/CAP guidelines and reinforced through national external quality assessment programs [23]. Variability in HER2 testing, caused by suboptimal tissue processing and the use of nonvalidated antibodies, has been well documented in Indian studies, underscoring the need for national quality assurance [23]. Standardized HER2 scoring and centralized confirmatory testing remain crucial to avoid misclassification and missed opportunities for therapies such as T-DXd. Although HER2 testing protocols are standardized in many tertiary centers, the reporting of HER2-low and ultralow categories remains inconsistent. This underscores the need for ongoing training and capacity building among pathologists to ensure accurate classification and treatment selection. AI-assisted HER2 IHC scoring demonstrated high concordance (up to 91.3%) with manual pathologist assessments across multi-institutional datasets while reducing scoring time by 50% [104]. These findings reflect analytical concordance and workflow efficiency; prospective studies evaluating the association of AI-assisted scoring with treatment selection and clinical outcomes are needed to further establish its role in standardizing HER2-low diagnostics.

Scaling up the regional hub-and-spoke model could facilitate equitable access to advanced molecular diagnostics such as HER2 IHC/FISH and NGS [47]. The DHR-ICMR Advanced Molecular Oncology Diagnostic Services (DIAMOnDS) is a pilot project initiated under the 15th Finance Commission (2020-2025) by the Department of Health Research. It aims to establish zonal molecular oncopathology laboratories across India to provide free, advanced diagnostic services for patients with cancer. These laboratories also support basic, translational, and clinical research in oncology. The initiative addresses the need for timely and accurate cancer diagnostics, especially in underserved regions, and promotes integrated diagnostics and research in a single setting [105]. To improve timely stratification, access to initial and repeat biomarker testing, including BRCA1/2, PD-L1, and NGS-based assays, should be integrated into routine care pathways. Coverage of these diagnostics under government schemes remains inconsistent, often requiring out-of-pocket expenditure or philanthropic support. Addressing these gaps is essential for equitable precision care.

To improve affordability and early detection, subsidized HER2 and BRCA testing should be implemented in alignment with ICMR’s 2023 guidelines [26,106].

Encouraging early referrals to tertiary or specialized centers when biomarker-directed care is anticipated could improve diagnostic accuracy and facilitate timely treatment selection [107]. National public awareness campaigns highlighting the importance of timely biomarker testing, especially in HER2-low and HR+/HER2- disease, are needed to educate patients and caregivers.

Clinician Engagement and Research Expansion

Clinicians have a pivotal role in translating policy into practice. Comprehensive biomarker profiling, including ER, PR, HER2, PD-L1, and BRCA1/2, should be performed at diagnosis and integrated into standard care pathways [108]. Clinicians should also link eligible patients to access programs or philanthropic initiatives that provide subsidized therapies, improving adherence and outcomes among under-resourced populations [36,109].

CME programs focused on HER2-low and HR+/HER2- classifications and the appropriate use of novel therapies can empower oncologists, particularly in tier-2 cities and rural areas. Parallel public awareness initiatives can encourage timely biomarker testing and treatment initiation.

At a national level, establishing an mBC registry would support real-time monitoring of disease patterns, treatment uptake, and survival outcomes. This data infrastructure would inform health policy decisions and promote India-specific clinical research.

Real-world data from oncology centers in India suggest that when guideline-based therapies such as trastuzumab and CDK4/6 inhibitors are accessible, clinical outcomes can approximate those reported in phase III trials. For example, CDK4/6 inhibitor-based regimens in HR+/HER2- mBC have demonstrated real-world median PFS of 20-27 months and objective response rates of up to 80%, aligning closely with PALOMA-2 trial outcomes [67]. However, these benefits are not universal due to disparities in access, especially in rural and public healthcare settings, where late-stage presentation and suboptimal treatment adherence are common [40]. Economic evaluations reveal that while guideline-based therapies can improve survival and quality-adjusted life years (QALYs), their cost often exceeds India’s willingness-to-pay thresholds, making them less cost-effective unless short-duration regimens are used [110]. Furthermore, limited real-world data on hospital admissions and toxicity-related healthcare burden indicate increased strain on the system when diagnosis is delayed or treatment is incomplete [25]. Collectively, these findings underscore the dual challenge of improving access to evidence-based therapies while addressing the financial and infrastructural limitations of the Indian oncology landscape.

Improving outcomes in mBC requires an integrated strategy that expands biomarker testing, embeds psycho-oncology into routine care, strengthens regional workforce training, and reduces stigma. Nonpharmacologic interventions such as yoga, meditation, and supportive psychotherapy have shown benefits in reducing depression and improving adherence [111].

Clinical trials are essential to advancing cancer care, yet participation in India, especially for mBC, remains low due to limited awareness and deep-rooted social stigma [112]. Despite initiatives like ICMR’s Phase I Network to build trial capacity and infrastructure [113], many patients are unaware of the benefits or fear discrimination. Patient advocacy groups play a transformative role in this space by fostering trust, sharing survivor stories, and encouraging informed decision-making [114]. Tackling stigma through education and community engagement is critical to normalizing trial participation and empowering patients to access innovative treatments [115]. Promoting ethically conducted, patient-centered clinical trials across India is essential to enhancing access to innovative cancer therapies.

A unified approach combining clinical precision, psychosocial support, and system-level reform can reduce emotional suffering, prevent treatment abandonment, and enable equitable access to life-extending therapies across India. Strategic alignment between clinicians, policymakers, and public-private stakeholders will be essential to ensuring that the benefits of innovative mBC therapies are equitably accessible, regardless of geographic or socioeconomic barriers.

Figure 1 and Table 2 summarize the diagnostic and therapeutic gaps, as well as actionable strategies, for improving targeted therapy access in mBC in the Indian context.

**Figure 1:**
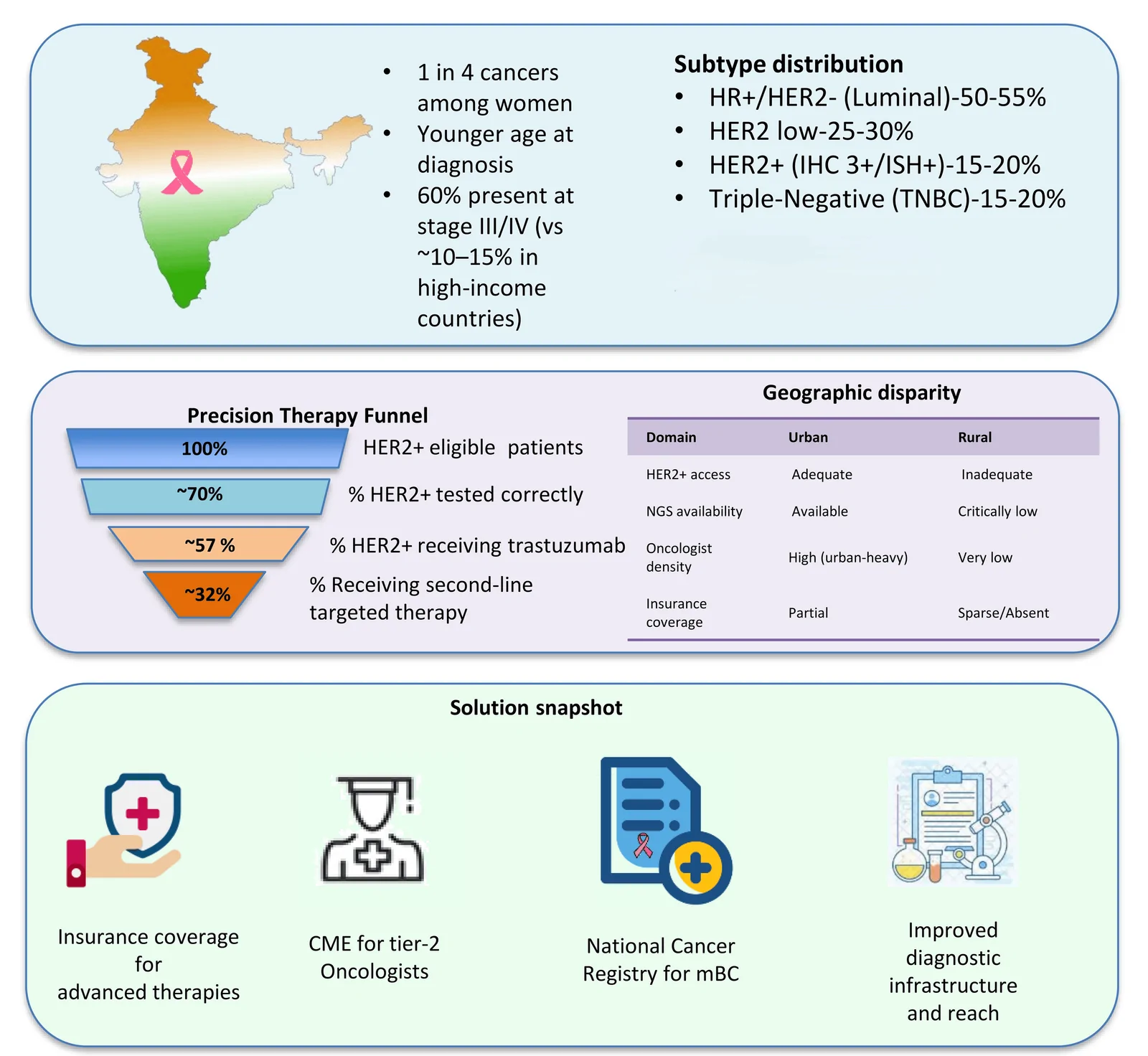
Bridging the precision gap in advanced/metastatic breast cancer care in India The infographic illustrates key challenges and potential intervention points in the management of advanced and metastatic breast cancer (mBC) in India. It highlights epidemiological patterns, including a younger age at diagnosis and a high proportion of late‑stage presentation, along with the distribution of major breast cancer subtypes. The precision therapy funnel demonstrates attrition across sequential steps, from human epidermal growth factor receptor 2 (HER2) eligibility and testing to receipt of targeted therapies. Marked geographic disparities between urban and rural settings are shown across access to HER2 testing, next‑generation sequencing, oncologist availability, and insurance coverage. Potential strategies to address these gaps include strengthening standardized HER2 testing, improving access to advanced targeted therapies, enhancing continuing medical education for tier‑2 oncology providers, establishing a national registry for mBC, and expanding diagnostic infrastructure to support more equitable delivery of precision oncology care [2–5,16,20,36,91]. Credit: This is an original illustration created by the authors using Microsoft PowerPoint (Microsoft Corporation, Redmond, Washington) and free-to-use elements from Pixabay, Flat Icons, Stockes Design, Shutterstock, and Canva.

**Table 2:** Summary of key barriers and strategic solutions for precision care for advanced/metastatic breast cancer in India AB-PMJAY: Ayushman Bharat Pradhan Mantri Jan Arogya Yojana; ADC: antibody-drug conjugate; AI: artificial intelligence; ASCO: American Society of Clinical Oncology; CAP: College of American Pathologists; CME: continuing medical education; EML: Essential Medicines List; ESMO: European Society for Medical Oncology; FISH: fluorescence in situ hybridization; HER2: human epidermal growth factor receptor 2; HER2-low: low-level HER2 expression (IHC 1+ or 2+ and ISH-negative); HER2-ultralow: ultralow HER2 expression (IHC 0 with faint staining in ≤10% of cells); IHC: immunohistochemistry; ISH: in situ hybridization; mBC: metastatic breast cancer; NCCN: National Comprehensive Cancer Network; NGS: next-generation sequencing; T-DXd: trastuzumab deruxtecan. Data from references [18,23,39,43,50,56,63,64,76,78,82,86,89–91,93,96].

Domain	Barrier	Strategic Solutions
Diagnostics	HER2-low/ultralow classification inconsistencies Lack of standardized IHC/FISH/NGS protocols Limited rural access to biomarker testing	National HER2 testing standardization (ASCO/CAP aligned); AI-based diagnostic support; regional testing hubs
Treatment access	Delayed availability of novel agents like T-DXd Limited public sector supply Out-of-pocket costs	AB-PMJAY and state insurance expansion to cover ADCs Public–private partnerships for affordable drug access
Workforce	Urban-centric oncologist distribution Low pathologist-to-patient ratio Limited awareness of HER2-low as a targetable subgroup	CME programs on HER2-low and precision oncology Incentives for rural oncology deployment
Economic impact	High out-of-pocket expenditure and catastrophic health spending Poor insurance penetration	Price caps on essential targeted therapies Inclusion of HER2-targeted agents in national schemes
Psychosocial burden	High depression, anxiety, and stigma Limited mental health support in cancer care	Integration of psycho-oncology services Support groups and non-pharmacologic therapies (e.g., yoga, counseling)
Policy and governance	Delays in regulatory approvals Weak adoption of global guidelines (e.g., NCCN, ESMO) Lack of mBC data infrastructure	Fast-track approvals for breakthrough therapies Nationwide mBC registry; India-adapted clinical pathways

Limitations

This narrative review has certain limitations. The evidence base was heterogeneous and included observational studies, retrospective studies, registry analyses, conference abstracts, guidelines, policy documents, and international clinical trial data. Much of the available Indian evidence was derived from single-center and retrospective studies, which may limit the generalizability of the findings. In addition, no formal risk-of-bias assessment or evidence-quality grading was performed, consistent with the narrative review design, which limits the ability to assess the overall strength of the evidence. Comparisons between Indian and global data should also be interpreted with caution because of differences in healthcare systems, diagnostic access, treatment availability, and outcome reporting.

## Conclusions

To align breast cancer care in India with global standards, a coordinated and timely response is crucial. It is imperative to address diagnostic limitations, economic barriers, and systemic inequities. Standardized biomarker testing, broader access to targeted therapies, multidisciplinary care integration, along with comprehensive physician and patient education, will help bridge the existing gaps between innovation and implementation. Precision medicine should not remain a privilege; it needs to evolve into accessible care for all, ensuring that every patient, regardless of geography or socioeconomic status, receives timely and targeted treatment, thereby maximizing outcomes while minimizing avoidable suffering.
